# Comparative Metabolomic Analysis of the Nutrient Composition of Different Varieties of Sweet Potato

**DOI:** 10.3390/molecules29225395

**Published:** 2024-11-15

**Authors:** Xiaolin Wan, Xiuzhi Wang, Qiang Xiao

**Affiliations:** Hubei Key Laboratory of Biological Resources Protection and Utilization, Hubei Minzu University, Enshi 445000, China; 202230369@hbmzu.edu.cn (X.W.); 202330416@hbmzu.edu.cn (X.W.)

**Keywords:** sweet potato, untargeted metabolomics, amino acids, organic acids, lipids

## Abstract

Sweet potatoes are rich in amino acids, organic acids, and lipids, offering exceptional nutritional value. To accurately select varieties with higher nutritional value, we employed liquid chromatography–tandem mass spectrometry (LC-MS/MS) to analyze the metabolic profiles of three types of sweet potatoes (white sweet potato flesh, BS; orange sweet potato flesh, CS; and purple sweet potato flesh, ZS). When comparing CS vs. BS, ZS vs. BS, and ZS vs. CS, we found differences in 527 types of amino acids and their derivatives, 556 kinds of organic acids, and 39 types of lipids. After excluding the derivatives, we found 6 amino acids essential for humans across the three sweet potatoes, with 1 amino acid, 11 organic acids, and 2 lipids being detected for the first time. CS had a higher content of essential amino acids, while ZS had a lower content. Succinic acid served as a characteristic metabolite for ZS, helping to distinguish it from the other two varieties. These findings provide a theoretical basis for assessing the nutritional value of sweet potatoes and setting breeding targets while facilitating the selection of optimal varieties for food processing, medicine, and plant breeding.

## 1. Introduction

Sweet potato (*Ipomoea batatas* (L.) Lam.) is both an environmentally friendly crop and a key crop for food security and is widely grown in countries around the world [1,2]. The global annual production of sweet potatoes exceeds 90 million tons, with Asia accounting for approximately 71.1% of production, of which China is the most significant contributor [3]. Sweet potatoes are rich in nutrients and functional bioactive compounds. The former include starch, polyphenols, and carotenoids, making sweet potatoes an excellent energy source [4]. The latter include organic acids and lipids, which positively maintain balance within plants or the human body [5]. Amino acids in food provide essential nutrients for protein synthesis and participate in biochemical reactions in the human body [6]. Previous studies have found that common and critical nutrients, such as amino acids and organic acids, determine the quality of many foods [7]. For example, the level of amino acid metabolites in sweet potato leaves affects their taste [8]. Studies have also proven that plant-derived organic acids prevent chronic diseases like osteoporosis and obesity, treat metabolic acidosis, and regulate microbial metabolism in the human colon [9,10]. Different varieties of sweet potatoes have varying nutritional and flavor characteristics, and consumer preferences also vary. Despite their growing popularity, researchers have not yet thoroughly studied the nutritional components of sweet potatoes.

Untargeted metabolomics is an analytical method that can identify all small molecule metabolites in biological samples [11]. The food sector has widely applied this technology in recent years [12,13]. For instance, researchers have analyzed the fermentation process of *Crocus sativus* L. using untargeted metabolomics, discovering that the metabolic pathways of anthocyanins and amino acids are associated with their biosynthesis and general metabolic processes, respectively [14]. A study of the different metabolites in *Ophiocordyceps sinensis* (cultivated *O. sinensis* and wild *O. sinensis*) also showed that cultivated *O. sinensis* has higher amounts of amino acids, carbohydrates, and phenolic acids than wild *O. sinensis*. On the other hand, wild *O. sinensis* has much higher amounts of nucleosides and nucleotides [15]. Studies based on this technique have also found that the dissociation of membrane structures is associated with the browning of fresh-cut potatoes [16] and that the aging of black garlic provides new insights into food functionality and toxicological studies [17]. A broad-targeted metabolomics analysis of sweet potatoes showed that phenolic compounds are most common when the plants are stressed by drought and have many flavonoids [18]. Based on untargeted metabolomics, studies have reported that delphinidin, petunidin, and rosinidin are key metabolites for the purple pigmentation in purple sweet potatoes [19]. This technology has revealed the vast structural diversity of anthocyanins and flavonoids in purple and orange-fleshed sweet potatoes [20]. It has also been found that the accumulation of paeoniflorin and delphinidin compounds may be one of the main reasons for the purple coloration of sweet potato pulp [21]. The classification of four sweet potato varieties using near-infrared spectroscopy and gas chromatography–mass spectrometry revealed that amino acids and organic acids are key metabolites for tuber part differentiation and varietal specificity [22]. However, no research has utilized untargeted metabolomics to explore the differential metabolism of amino acids, organic acids, and lipids in the flesh of different sweet potato varieties.

Hence, in this study, we employed untargeted metabolomics to analyze the differential metabolites of amino acids, organic acids, and lipids in various sweet potato varieties. The goal was to understand the metabolic differences between these compounds in the different varieties of sweet potatoes, allowing for better selection and differentiation among the three types. In this study, we used multivariate statistical analyses to analyze the differential metabolites: these included principal component analysis (PCA), orthogonal partial least squares discriminant analysis (OPLS-DA), hierarchical clustering analysis (HCA), and Kyoto Encyclopedia of Genes and Genomes (KEGG) pathway analysis. Our findings provide a theoretical basis for evaluating the nutritional value of sweet potatoes and help identify the optimal varieties based on their applications in food processing, medicine, and plant breeding programs.

## 2. Results

### 2.1. Comparison of the Major Nutrient Metabolites of Three Sweet Potato Species

**Figure 1:**
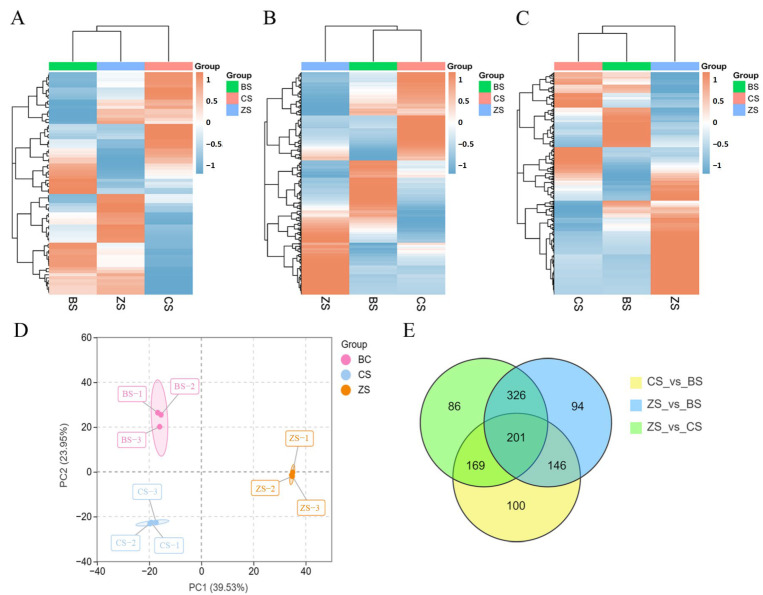
HCA, PCA, and Venn diagrams for the three types of sweet potatoes. (**A**) HCA diagram for lipids. (**B**) HCA diagram for organic acids. (**C**) HCA diagram for amino acids and their derivatives. Columns in the HCA diagram represent each variety of sweet potato, and rows represent each metabolite. Orange indicates a relatively high metabolite abundance, while blue indicates a relatively low abundance. (**D**) PCA score plot for the three types of sweet potatoes. (**E**) Venn diagram of the differential metabolites in the three comparison groups.

To better differentiate between different sweet potato varieties, we compared the accumulation patterns of amino acids and their derivatives, organic acids, and lipids in different sweet potato varieties. A cluster analysis showed that 73 lipid metabolites were detected in the sweet potatoes, including 58 free fatty acids (FFAs), 10 lysophosphatidylcholines (LPCs), 3 lysophosphatidylethanolamines (LPEs), and 2 glycerides (Figure 1A, Table S1). The relative content of total lipid metabolites was highest in BS and lowest in CS. ZS was enriched by a large number of FFA metabolites, including FFA (16:0), FFA (14:0), FFA (11:0), FFA (16:1), and FFA (17:0). While the relative contents of FFA (18:0) and FFA (18:2) were higher in BS, the relative contents of FFA (11:1) and FFA (18:1) were higher in CS. In CS, the relative abundance of amino acids and their derivatives was the highest (Figure 1B, Table S1). We detected six human essential amino acids in the different varieties of sweet potatoes and L-valine, L-tryptophan, L-phenylalanine, methionine, and L-lysine were highest in CS. L-isoleucine content was the highest in BS. Furthermore, we discovered that the essential amino acid content in all three sweet potatoes presented the following order: L-valine > L-tryptophan > L-isoleucine > L-phenylalanine > methionine > L-lysine. The relative abundance of organic acid metabolites was the highest in ZS (Figure 1C, Table S1). We found that the top 10 organic acids among the three sweet potato species included 1,3,4,5-tetrahydroxycyclohexanecarboxylic acid, D-malic acid, adipic acid, and 3-sialyl-N-acetyllactosamine.

### 2.2. Multivariate Statistical Analysis

In this study, a total ion chromatogram (TIC) showed the relative abundance of these metabolites (Figure S1). Furthermore, we looked at the three metabolite profiles using principal component analysis (PCA) and hierarchical cluster analysis (HCA) to learn more about the different types of amino acids and their derivatives, organic acids, and lipid metabolites found in the three sweet potato tubers. PCA reflects the overall metabolic differences between groups and the degree of variability among samples within groups. Principal component 1 (PC1) and component 2 (PC2) contributed 39.53% and 23.95% of the variance, respectively. In addition, the three sweet potato varieties displayed significant differences (Figure 1D).

HCA categorized the three metabolites into three significant clusters based on changes in their relative contents, with class 1 metabolites in BC, class 2 metabolites in CS, and class 3 metabolites in ZS showing the highest accumulation. All three sweet potato species showed a significant clustering of metabolites (Figure S2A). Each group was subjected to three replicates, demonstrating sufficient reproducibility and high data reliability. These findings indicated significant differences in the three samples’ metabolic profiles.

### 2.3. Identification of Differential Metabolites Among the Three Sweet Potato Varieties

**Figure 2:**
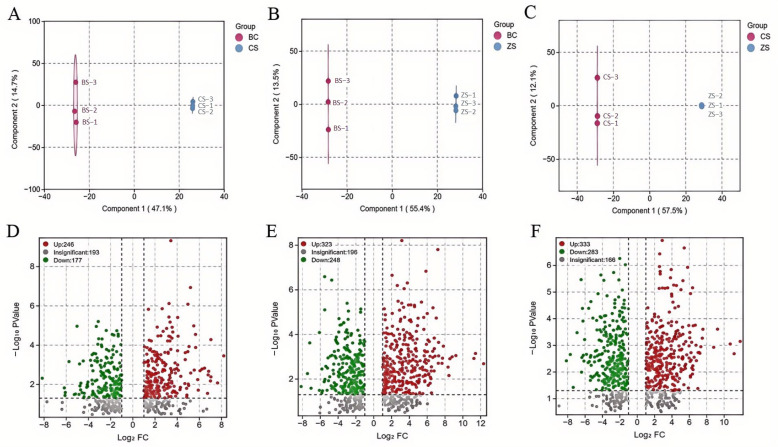
Differential metabolite analysis of the three sweet potato species. The score plots for (**A**) BS vs. CS, (**B**) BS vs. ZS, and (**C**) CS vs. ZS were derived using orthogonal partial least squares discriminant analysis (OPLS-DA). The volcano plots for (**D**) BS vs. CS, (**E**) BS vs. ZS, and (**F**) CS vs. ZS showed different levels of metabolites.

In order to examine the most significant changes in amino acids and their derivatives, organic acids, and lipid metabolism among the three sweet potato varieties, we used Venn diagrams to illustrate the shared differential metabolites among the comparison groups (CS vs. BS, ZS vs. BS, and ZS vs. CS) (Figure 1E, Table S2). In total, 201 common metabolites were identified. We also conducted an OPLS-DA analysis on the three sweet potato varieties. The results showed a clear separation between different groups (Figure S2B). We used OPLS-DA to identify the variables responsible for the differences among the three groups. We performed pairwise comparisons based on the OPLS-DA model to evaluate the three metabolites in the sweet potatoes, identifying differences between BS and CS (R2X = 0.618, R2Y = 1, Q2 = 0.93), between BS and ZS (R2X = 0.688, R2Y = 1, Q2 = 0.96), and between CS and ZS (R2X = 0.697, R2Y = 1, Q2 = 0.974) (Figure S3). The mean Q2 values for all comparisons exceeded 0.9, indicating the stability of these models. The OPLS-DA score plots effectively separated the three comparison groups, paving the way for a further VIP analysis to pinpoint differentially accumulated metabolites (Figure 2A–C).

Additionally, we conducted differential metabolite screening for all the annotated three types of metabolites based on FC, *p*-value, and VIP scores. The results were visually presented as volcano plots, with the criteria were set as FC ≥ 2 or ≤ 0.5, *p* < 0.05, and VIP ≥ 1. To summarize, there are 423 significantly different metabolites between CS and BS (246 upregulated, 177 downregulated) (Figure 2D). Among them, there are 217 amino acids and their derivatives (121 upregulated, 96 downregulated), 180 organic acids (113 upregulated, 67 downregulated), and 26 lipids (12 upregulated, 14 downregulated). Between ZS and BS, there are 571 metabolites (323 upregulated, 248 downregulated) (Figure 2E). Among them, there are 276 amino acids and their derivatives (125 upregulated, 151 downregulated), 280 organic acids (191 upregulated, 89 downregulated), and 15 lipids (7 upregulated, 8 downregulated). Between ZS and CS, there are 616 metabolites (333 upregulated, 283 downregulated) (Figure 2F). Among them, there are 292 amino acids and their derivatives (120 upregulated, 172 downregulated), 303 organic acids (202 upregulated, 101 downregulated), and 21 lipids (11 upregulated, 10 downregulated). The comparison between CS and ZS showed the highest number of differential metabolites.

K-means cluster analysis is a commonly used, unsupervised analytical method that groups samples or metabolites based on their characteristics. We scaled the relative contents of the three metabolites identified according to the screening criteria in the comparison group (by unit variance) and then subjected them to a k-means cluster analysis to investigate the trends in the relative contents in sweet potatoes with different varieties. Then, we used a k-means cluster analysis to look at how the amounts of these metabolites changed over time in the sweet potatoes that looked different. Through the analysis, we discovered 10 distinct trends among the 1122 differential metabolites from the three different varieties. Of these, 298 metabolites in Clusters 1 and 6 belonged to the ZS group and were more abundant than the other metabolites. In Cluster 9, 85 metabolites from the CS group were more abundant than the other metabolites (Figure S4).

### 2.4. KEGG Annotation and Enrichment Analysis of Differential Metabolites

**Figure 3:**
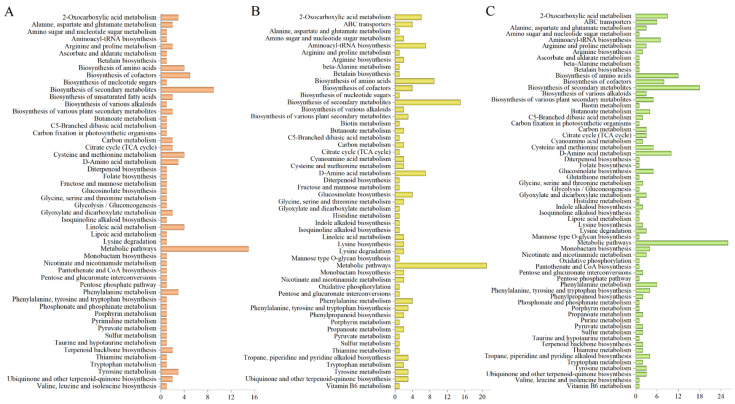
KEGG annotations. (**A**) Differential accumulation enrichment results of the three metabolites for CS vs. BS, (**B**) ZS vs. BS, and (**C**) ZS vs. CS. Horizontal coordinates represent the number of differential metabolites enriched in a pathway (orange represents the number of differential metabolites between CS vs. BS, yellow represents the number of differential metabolites between ZS vs. BS, and green represents the number of differential metabolites between ZS vs. CS); vertical coordinates represent the KEGG pathway names. The horizontal coordinates indicate the number of metabolites annotated in each pathway.

This study annotated and enriched the differential metabolites of each comparison group and divided them into different KEGG pathways. According to the KEGG database, differential metabolites in the CS vs. BS, ZS vs. VS, and ZS vs. CS groups were enriched in 52, 53, and 63 pathways, respectively. In CS vs. BS, the three metabolites were mainly enriched in “metabolic pathways”, “biosynthetic of secondary metabolites”, “biosynthetic of cofactors”, “cysteine and methionine metabolism”, “biosynthetic of amino acids”, “linoleic acid metabolism”, “2-oxocarbolic acid metabolism”, “D-amino acid metabolism”, “tyrosine metabolism”, and “phenylalanine metabolism” (Figure 3A). In ZS vs. BS, the three metabolites were mainly enriched in “metabolic pathways”, “biosynthetic of secondary metabolites”, “biosynthetic of amino acids”, “D-amino acid metabolism”, “aminoacyl tRNA biosynthesis”, “2-oxocarbolic acid metabolism”, “ABC transporters”, “phenylalanine metabolism”, “glucosinolate biosynthesis”, and “photosynthesis of cofactors” (Figure 3B). In ZS vs. CS, the three metabolites were mainly enriched in “metabolic pathways”, “biosynthetic of secondary metabolites”, “biosynthetic of amino acids”, “D-amino acid metabolism”, “2-oxocarbolic acid metabolism”, “biosynthetic of cofactors”, “aminoacyl tRNA biosynthesis”, “ABC transporters”, “phenylalanine metabolism”, “cysteine and methionine metabolism”, and “phenylalanine, tyrosine, and tryptophan biosynthesis” (Figure 3C).

### 2.5. Significant Differences in Each Comparison Group for the Three Metabolites

To explore the critical differential metabolites in the three comparison groups, we produced Venn diagrams to depict the relationships between the combined differential metabolites of CS vs. BS, ZS vs. BS, and ZS vs. CS. A total of 201 overlapping differential metabolites were identified in the three two-by-two comparisons. The same differential metabolites in each comparison group are shown in Table S3. Compared to the other two sweet potatoes, BS was downregulated for 71 substances, of which a total of 39 were significantly different (11 amino acids and their derivatives, 27 organic acids, and 1 lipid), and upregulated for 59 substances, of which a total of 49 were significantly different (38 amino acids and their derivatives, 10 organic acids, and 1 lipid). Compared to the other two sweet potatoes, CS was downregulated for 41 substances, of which 17 were significantly different (5 amino acids and their derivatives, 12 organic acids), and upregulated for 60 substances, of which 52 were significantly different (28 amino acids and their derivatives, 21 organic acids, 3 lipids). In ZS, 89 substances were downregulated compared to the other two sweet potatoes, of which a total of 58 were significantly different (44 amino acids and their derivatives, 12 organic acids, and 2 lipids), and 82 substances were upregulated, of which a total of 63 were significantly different (16 amino acids and their derivatives, 45 organic acids, and 2 lipids).

Among the 201 overlapping differential metabolites, only four metabolites—SureCN33428, hydrogenobyrinate diamide, 4-(beta-D-glucosyloxy) benzoic acid, and N-formylmethionine—were detected in seven KEGG metabolic pathways (metabolic pathways, biosynthesis of secondary metabolites, biosynthesis of cofactors, cyanide and methionine metabolism, ubiquinone and other terpenoid-quinone biosynthesis, porphyrin metabolism, and diterpenoid biosynthesis) (Table S1).

**Figure 4:**
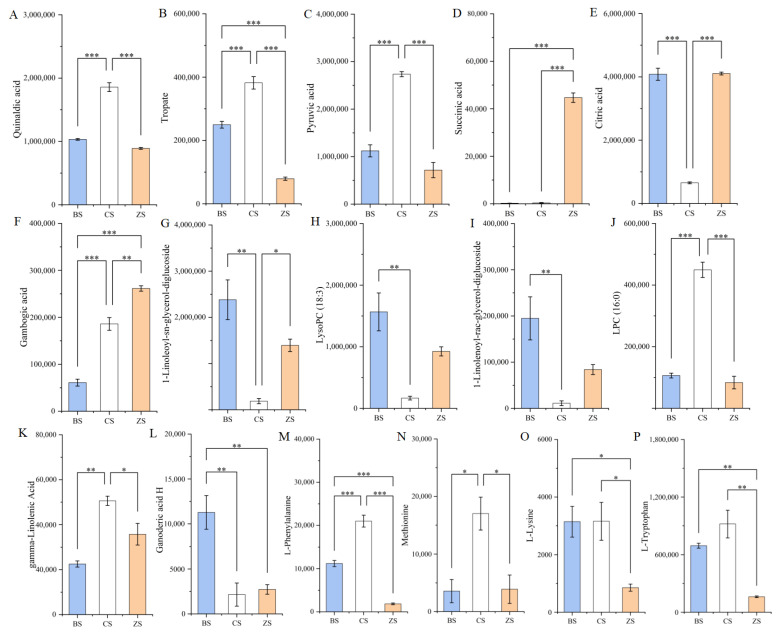
Peak area plots of several amino acids, organic acids, and lipids. (**A**–**F**): peak area values for several common organic acids; (**G**–**L**): peak area values for several common lipids; (**M**–**P**): peak area values of four human essential amino acids. * represents *p* ≤ 0.05; ** represents *p* ≤ 0.01; *** represents *p* ≤ 0.001. Blue represents BS, white represents CS, and orange represents ZS.

Among the differential metabolites of amino acids and their derivatives, we found four human essential amino acids exhibiting significant differences, as shown by their peak area values in Figure 4M–P. Methionine content in CS is significantly higher than in BS and ZS, while there is no significant difference between BS and ZS. L-lysine content differs significantly between BS and ZS, but both BS and ZS show no significant difference compared to CS. There is no significant difference in L-tryptophan and L-phenylalanine content between BS and CS, but both BS and CS show significant differences compared to ZS. Many different forms of citric acid, pyruvic acid, quinaldic acid, gambogic acid, tropate, succinic acid, and quinic acid were found among the different metabolites of organic acids. Specifically, the citric acid content in BS is 6.24 times higher than in CS, and the citric acid content in ZS is 6.28 times higher than in CS. The pyruvic acid content in CS is 2.44 times higher than in BS and 3.82 times higher than in ZS. The succinic acid content in ZS is higher than in both BS and CS, being 202.04 times that of BS and 128.83 times that of CS. Additionally, the content of quinic acid derivatives (1,5-diferuloylquinic acid and 4-O-feruloyl-D-quinic acid) in ZS is higher than in both BS and CS. Figure 4A–F shows the peak area values of several common organic acids. In lipids, we found that the LPC 16:0 content in CS is significantly higher than in BS and ZS, while the LPC 18:1 content is significantly lower. The content of ganoderic acid derivatives, LysoPC 18:3, and 1-linolenoyl-rac-glycerol-diglucoside in BS is significantly higher than in CS and ZS. Figure 4 G–L shows the peak area values of several common lipids. Table S4 lists the FC and Log2 FC values for all differential metabolites.

## 3. Discussion

Sweet potato is becoming increasingly popular due to its wide variety and rich nutritional value. Non-targeted metabolomics analyses reveal the nutrient metabolites among different plant species [23]. Previous studies have used broadly targeted metabolomics techniques to identify 496 metabolites in white “Xushu 28”, orange “Xushu 34”, and purple “XuZishu 6” sweet potatoes. This amount included 74 amino acids and their derivatives and 86 organic acids, accounting for 14.92% and 17.34% of the total metabolites, respectively [24]. In this study, we performed an untargeted metabolomics analysis on three sweet potato varieties. We detected amino acids and their derivatives (797), organic acids (863), and lipids (73), which accounted for 17.92%, 19.41%, and 1.64% of the total metabolites. Our study revealed a more significant number and diversity of metabolites in the three sweet potatoes than previous studies.

Among the identified amino acids and their derivatives, the three sweet potatoes contained six (L-Isoleucine, L-valine, D-phenylalanine, methionine, L-lysine, and L-tryptophan) human essential amino acids. In previous studies, these essential amino acids have been found to counteract oxidative stress [25] and bioenhancement [26]. Notably, four (methionine, L-lysine, L-tryptophan, and L-phenylalanine) of the human essential amino acids were significantly different in the three sweet potatoes. The levels of human essential amino acids in ZS were all low, the levels of human essential amino acids in CS were all high, and the levels of L-phenylalanine in BS significantly differed from both CS and ZS. Screening for amino acids with a peak area mean value greater than 10,000, we detected L-theanine for the first time in sweet potatoes, which has been shown in previous studies to prevent colds and influenza by boosting immunity [27]. Among the organic acids, we found that the succinic acid content of ZS was 202.04 times higher than that of BS and 128.93 times higher than that of CS. In addition, there was no significant difference in succinic acid content between BS and CS. Therefore, we concluded that succinic acid could be used as a characteristic metabolite for judging ZS and the other two sweet potatoes. Citric acid, a common metabolite in sweet potatoes, had the lowest content in CS. Notably, screening the organic acids with peak areas greater than 10,000, we identified gambogic acid, adipic acid, 3,5-dihydroxybenzoic acid, 3-Oxooctadecanoic acid, 3-Hydroxyglutaric acid, 3-Furoic acid, 3-Hydroxyglutaric acid, 3-Furoic acid, and 3-Hydroxybutyric acid for the first time in sweet potatoes. Among these compounds, gambogic acid has been shown to have anticancer effects [28]. Among the organic acids with an average peak area of less than 10,000, lentinic acid, humic acid, N-acetyldjenkolic acid, and chorismic acid were also identified for the first time in sweet potatoes. Lentinic acid is a flavor precursor of shiitake mushrooms and can accelerate the metabolism of ethanol in the stomach and liver and inhibit the absorption of ethanol in the stomach and jejunum [29]. Chorismic acid is located at a critical branching point of the mangiferolic acid pathway. It is involved in the biosynthesis of various monocyclic and polycyclic aromatic metabolites, which can be used as targets for drug development [30]. We detected ganoderic acid H and gamma-linolenic acid in lipids for the first time. Ganoderic acid H is able to inhibit the growth and invasion of breast cancer cells by inhibiting the signaling of transcription factors AP-1 and NF-kappaB [31]. Supplementation with foods containing gamma-linolenic acid may reduce various inflammatory responses [32,33].

Each Convolvulaceae species is characterized by its unique metabolite accumulation. This study used HCA and total peak area analysis to show that the amounts of amino acids and their derivatives, organic acids, and lipids significantly differed between the different sweet potato types. Most of the amino acids and their derivatives were found in higher amounts in BS and CS, while the amounts of organic acid metabolites were higher in ZS, and lipids were higher in BS. These results suggest that differences in the three metabolites may exist among different sweet potato varieties, leading to different effects on the human body.

In addition, the results of the PCA showed significant differences among the three sweet potato varieties. The three comparison groups (CS vs. BS, ZS vs. BS, and ZS vs. CS) identified 1733 metabolites of amino acids, organic acids, and lipids and 1122 differential metabolites. In the three pairwise comparisons, there were 201 overlapping differential metabolites, of which amino acids, their derivatives, and organic acids accounted for 47.96% and lipids for 4.48%. Among these substances, we detected N-acetyldjenkolic acid in sweet potatoes for the first time, with ZS being 18.30-fold more abundant than BS and 1,5-Diferuloylquinic acid exhibiting antioxidant activity [34]. Furthermore, we discovered that many small peptides, formed by multiple amino acids in tandem, are present in amino acids and their derivatives. The human body may hydrolyze these small peptides into amino acids for absorption and utilization [35]. The analysis of KEGG metabolic pathways showed that the three comparison groups had 36 identical KEGG pathways. The examination of these pathways revealed the highest number of differential metabolites among the three groups. These pathways included the biosynthesis of secondary metabolites, cofactors, amino acids, phenylalanine metabolism, D-amino acid metabolism, and 2-oxocarboxylic acid metabolism. Identifying these differential metabolites would be helpful in the nutritional evaluation of different varieties and facilitate the selection of superior sweet potato varieties.

## 4. Materials and Methods

### 4.1. Plant Material

The Hubei Academy of Agricultural Sciences provided us with three sweet potato varieties: white sweet potato flesh “Chestnut Potato” (BS), orange sweet potato flesh “Watermelon Red No. 1” (CS), and purple sweet potato flesh “Purple Potato” (ZS). In the summer of 2022, we planted sweet potato plants in plastic root controllers containing 15 L of peat soil and vermiculite (1:8 ratio), after which we monitored the soil daily for wetness and rationed watering. During the harvest period, we harvested and immediately froze sweet potato flesh samples in liquid nitrogen. We stored the samples at −80 °C until we extracted the metabolites.

### 4.2. Dry Sample Extraction

We placed biological samples in a lyophilizer (Scientz-100F, Ningbo, China) using vacuum freeze-drying to remove moisture. The dried samples were then ground into powder using a grinder (MM 400, Retsch, Haan, Germany) at 30 Hz for 1.5 min. Utilizing an electronic balance, a 50 mg sample was carefully weighed out. Then, we added 1200 μL of 70% methanol aqueous solution internal standard extract (2-chlorophenylalanine) cooled to −20 °C. We vortexed the mixture for 30 s every 30 min, resulting in a total of 6 cycles. After centrifugation at 12,000 rpm for 3 min, the supernatant was collected and filtered through a 0.22 μm microporous membrane. We then stored the filtered solution in an injection vial for UPLC-MS/MS analysis.

### 4.3. Liquid Chromatography and Tandem Mass Spectrometry Conditions

An LC-MS system was utilized to process all samples according to standard machine protocols. The analytical setup included an ACQUITY UPLC HSS T3 column (1.8 µm, 2.1 mm × 100 mm) from Waters. The column temperature was maintained at 40 °C with a flow rate of 0.40 mL/min, and the injection volume was set to 4 μL. The solvent system consisted of water with 0.1% formic acid (solvent A) and acetonitrile with 0.1% formic acid (solvent B). A gradient program was used for sample measurement, starting with 95% A and 5% B. Over the course of 5 min, the gradient shifted linearly to 35% A and 65% B. In the following minute, it shifted to 1% A and 99% B, which was maintained for 1.5 min. Finally, the composition returned to 95% A and 5% B within 0.1 min and was held for 2.4 min.

Data acquisition was performed in information-dependent acquisition (IDA) mode using Analyst TF 1.7.1 software (Sciex, Concord, ON, Canada). The source parameters were configured as follows: ion source gas 1 (GAS1) at 50 psi, ion source gas 2 (GAS2) at 60 psi, and curtain gas (CUR) at 35 psi. The temperature was set to 550 °C, with declustering potential (DP) at 80 V for the positive mode and −80 V for the negative mode. The ion spray voltage floating (ISVF) was set at 5500 V for the positive mode and −4500 V for the negative mode. The TOF MS scan parameters were as follows: mass range of 50–1250 Da, accumulation time of 200 ms, and dynamic background subtraction activated. The product ion scan parameters were as follows: mass range of 50–1250 Da, accumulation time of 40 ms, and collision energy of 30 V in both positive and negative modes, with a collision energy spread of 15. The resolution was set to UNIT, with a charge state of 1, intensity of 100 cps, and exclusion of isotopes within 4 Da. Up to 12 candidate ions were monitored per cycle with a mass tolerance of 50 mDa.

### 4.4. Metabolite Identification and Quantification

Raw data were first converted to the mzXML format using Proteo Wizard. Peak extraction, alignment, and retention time correction were then performed with XCMS software. To ensure data quality, peaks with a missing rate above 50% in any sample set were excluded, and missing values were imputed using the KNN method. Peak areas were further corrected using the SVR method. Metabolite identification was carried out by matching the processed peaks against the Metware Biotechnology Company (Wuhan, China) proprietary database, as well as public and predictive libraries, utilizing the MetDNA approach. Substances with an identification composite score below 0.5 and a coefficient of variation (CV) less than 0.3 in the QC samples were selected for further analysis. Positive and negative ion modes were integrated to retain compounds with the highest identification confidence and the lowest CV values, generating a comprehensive data file for all samples. Metabolites were characterized using both primary and secondary MS data, and their relative abundance across samples was determined based on the chromatographic peak areas.

### 4.5. Statistical Analysis

We analyzed the metabolic data from each sample using HCA, PCA, and OPLS-DA to study the accumulation of varietal lipid-, organic acid-, and amino acid-specific metabolites. We displayed the HCA results for metabolites and samples using heatmaps and dendrograms. We plotted HCA using package R. Before performing HCA and PCA, we unit-variance-scaled the metabolite data. We then executed OPLS-DA models using the R package (Metabo Analyst R) version 1.0.1 to compare the metabolic profiles of the different sweet potato varieties. Metabolite data were log2 transformed to improve normality and mean centering before the OPLS-DA analysis. We performed a permutation test (200 permutations) to prevent overfitting. In the OPLS-DA models, variables with a projection (VIP) value ≥ 1 and an absolute log2 fold change (FC) value ≥ 1 were set as important for screening different metabolites. We displayed the number of differential metabolites using Venn diagrams.

### 4.6. KEGG Annotation and Metabolic Pathway Analysis of Differential Metabolites

We annotated the identified metabolites using the KEGG compound database (http://www.kegg.jp/kegg/compound/ (accessed on 23 February 2024)) and then mapped the annotated metabolites to the KEGG pathway database (http://www.kegg.jp/kegg/pathway.html (accessed on 25 February 2024)). We then entered the pathways that mapped significantly regulated metabolites into MSEA (Metabolite Set Enrichment Analysis), determining their significance by the *p*-value of the hypergeometric test.

## 5. Conclusions

This study employed untargeted metabolomics to systematically evaluate the metabolic profiles of amino acids and their derivatives, organic acids, and lipids in three sweet potato varieties, focusing on the differences in metabolites between these varieties. We detected a total of 1733 metabolites (797 amino acids and their derivatives, 863 organic acids, and 73 lipids), of which 1122 were differential metabolites (527 amino acids and their derivatives, 556 organic acids, and 39 lipids). For the first time, the amino acid L-theanine was identified, and the study confirmed variations in the contents of several essential amino acids for humans (such as methionine, L-lysine, L-tryptophan, and L-phenylalanine) among the different sweet potato varieties. We also found higher levels of essential amino acids in CS and lower levels in ZS. Regarding organic acids, succinic acid was a characteristic marker distinguishing ZS from the other varieties. Additionally, several organic acids, including gambogic acid, adipic acid, lentinic acid, and chorismic acid, were detected in sweet potatoes for the first time, each possessing significant functional properties. The lipid analysis also revealed the presence of ganoderic acid H and gamma-linolenic acid in sweet potatoes for the first time, both of which offer potential health benefits. By comparing the three sweet potato varieties, the study identified 201 overlapping differential metabolites, with amino acids and their derivatives and organic acids each accounting for 47.96% and lipids for 4.48%. Notably, the content of N-acetyldjenkolic acid in ZS was significantly higher than in the other varieties, with levels 18.30 times higher in ZS compared to BS, suggesting its potential as a marker for distinguishing sweet potato varieties. The KEGG pathway analysis revealed that these differential metabolites were primarily enriched in metabolic pathways, secondary metabolite biosynthesis, and amino acid biosynthesis. These pathways offer insights into the metabolic processes in sweet potatoes, and future research can further focus on these critical pathways. This study contributes to a better understanding of the metabolite composition of the three sweet potato varieties and provides breeders with a basis for evaluating and selecting varieties with beneficial nutritional and functional traits.

## Data Availability

The data are contained within the article.

## Supplementary Materials

The following supporting information can be downloaded at: https://www.mdpi.com/article/10.3390/molecules29225395/s1. Figure S1. Total ion chromatograms (TIC) of amino acids, organic acids and lipids in different varieties of sweet potato. Figure S2. Total HCA and OPLS-DA plots for three types of sweet potatoes. Figure S3. OPLS-DA model verification diagram. Figure S4. K-means clusters of the expression profiles of three sweet potatoes. Table S1. Amino acids, organic acids, and lipids in the flesh of three sweet potato varieties. Table S2. Overlapping differential metabolites in different comparison groups. Table S3. Identical differential metabolites in each comparison group. Table S4. FC and Log2FC values for all differential metabolites.
