# Supplementary material for: Comparative Metabolomic Analysis of the Nutrient Composition of Different Varieties of Sweet Potato
Source: Molecules. 2024 Nov 15;29(22):5395. doi: 10.3390/molecules29225395 (PMC11597878; doi:10.3390/molecules29225395)
Supplement: Supplementary file 1 [file molecules-29-05395-s001.zip › Figure S1-S4.pdf]

Comparative Metabolomic Analysis of the Nutrient Composition of Different Varieties of Sweet Potato

Xiaolin Wan, Xiuzhi Wang, Qiang Xiao*

[1] These authors contributed equally to this work.

Hubei Key Laboratory of Biological Resources Protection and Utilization, Hubei Minzu University, Enshi 445000, China

***Corresponding author:**

*Corresponding author:

Professor Xiao Qiang

39 Xueyuan Road, Enshi City, Hubei Province, Hubei Minzu University, Postal Code 445000

Phone: +86 15171698508; Email: 1992022@hbmzu.edu.cn

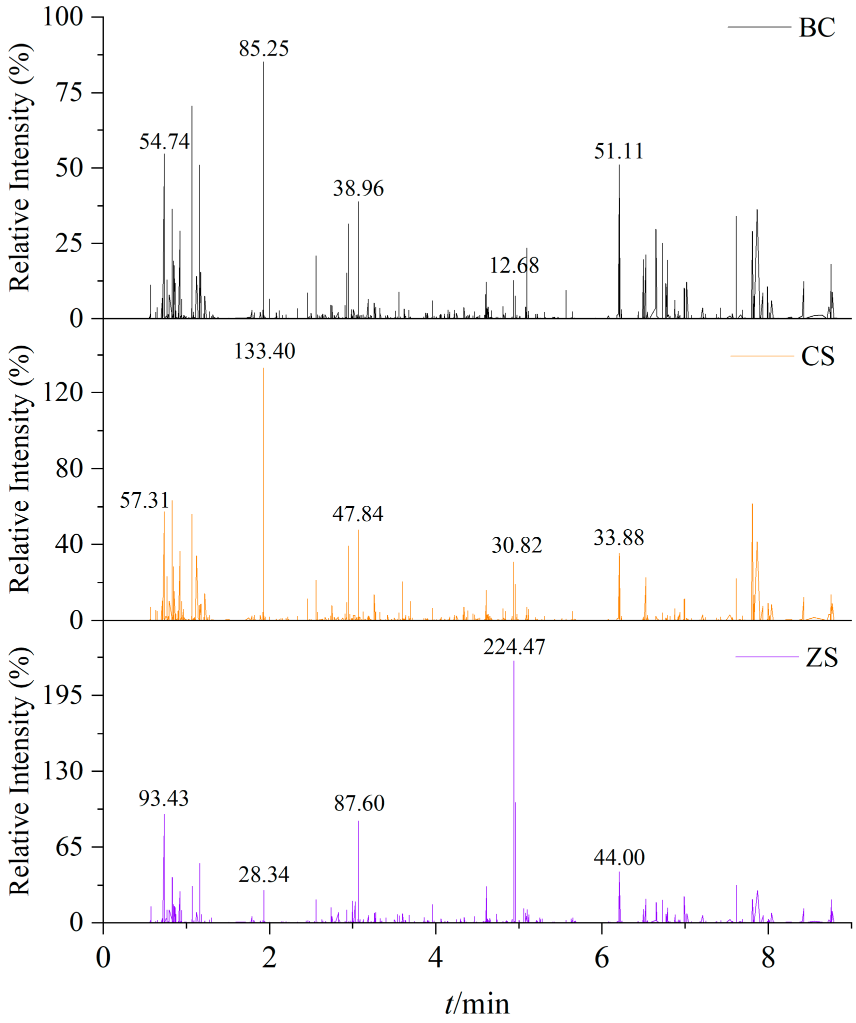

Figure S1. Total ion chromatograms (TIC) of amino acids, organic acids and lipids in different varieties of sweet potato.

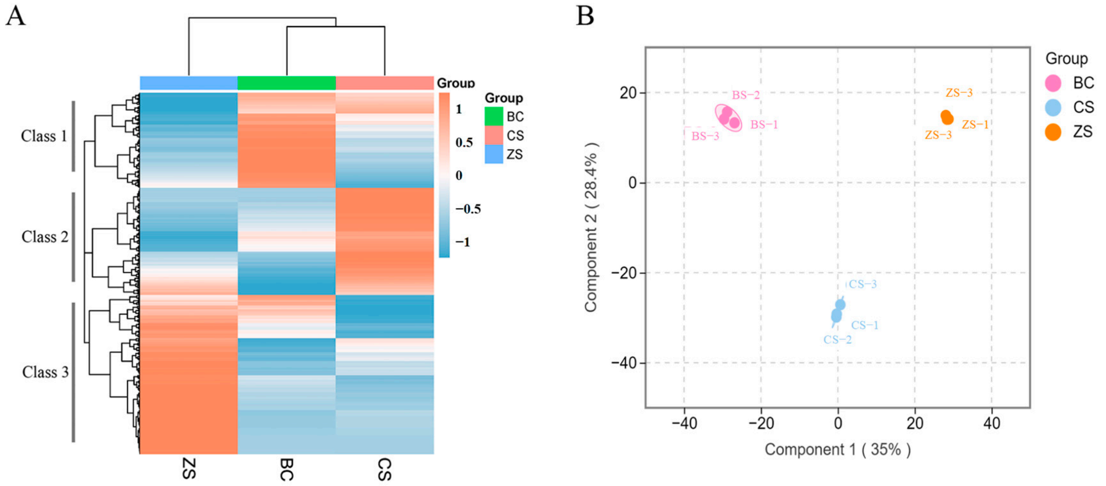

Figure S2. Total HCA and OPLS-DA plots for three types of sweet potatoes. (A) HCA plot for all metabolites; (C) OPLS-DA score plot.

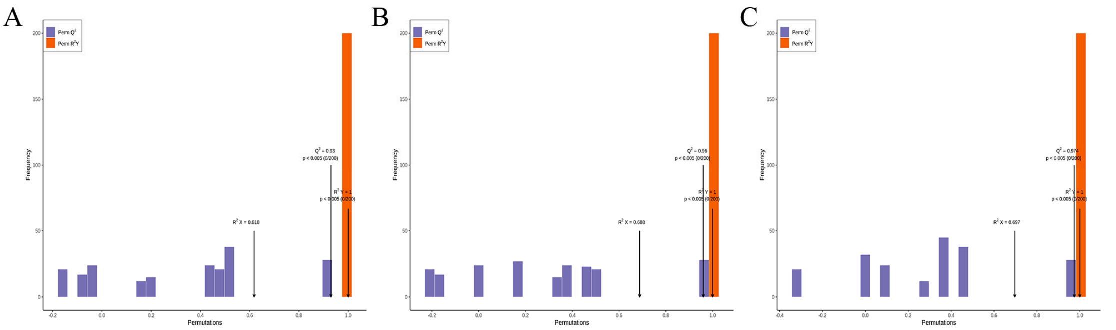

Figure S3. OPLS-DA model verification diagram. **(A)** ‘BS’ and ‘CS’; **(B)** ‘BS’ and ‘ZZS’; **(C)** ‘CS’ and ‘ZS’.

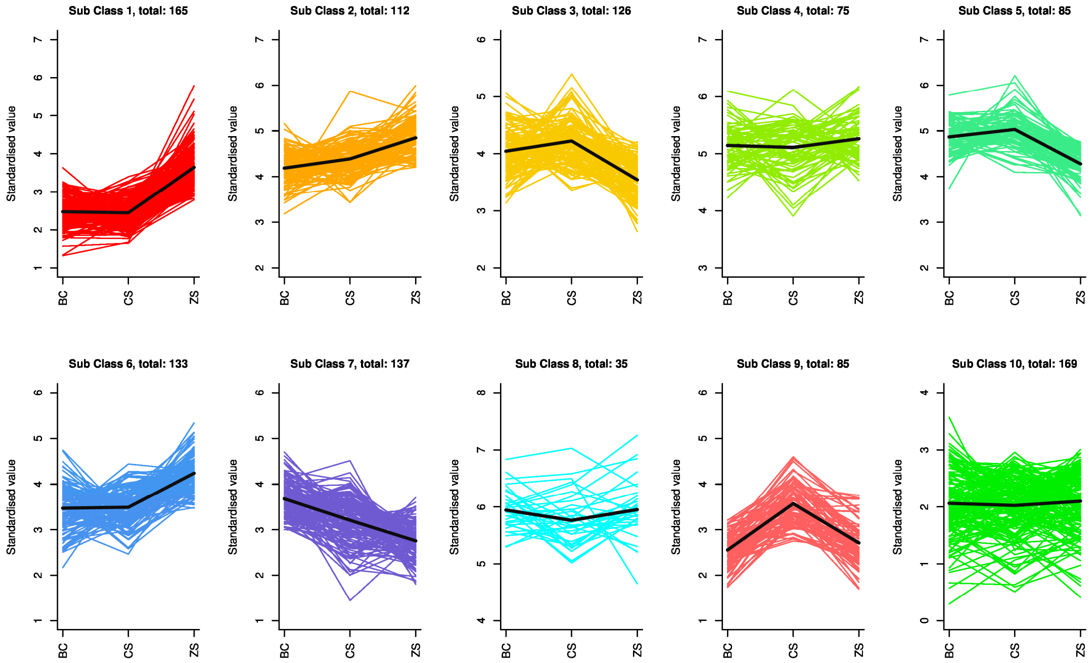

Figure S4. K-means clusters of the expression profiles of three sweet potatoes. The y-axis represents the normalized metabolite content and the x-axis represents the different samples.
